# The ATP-gated P2X_1_ ion channel contributes to the severity of antibody-mediated Transfusion-Related Acute Lung Injury in mice

**DOI:** 10.1038/s41598-019-41742-9

**Published:** 2019-03-26

**Authors:** Marie-Belle El Mdawar, Blandine Maître, Stéphanie Magnenat, Christian Gachet, Béatrice Hechler, Henri de la Salle

**Affiliations:** Université de Strasbourg, INSERM, Etablissement Français du Sang (EFS) Grand Est, BPPS UMR_S 1255, Fédération de Médecine Translationnelle de Strasbourg (FMTS), F-67000 Strasbourg, France

## Abstract

The biological responses that control the development of Transfusion-Related Acute Lung Injury (TRALI), a serious post-transfusion respiratory syndrome, still need to be clarified. Since extracellular nucleotides and their P2 receptors participate in inflammatory processes as well as in cellular responses to stress, we investigated the role of the ATP-gated P2X_1_ cation channel in antibody-mediated TRALI. The effects of NF449, a selective P2X1 receptor (P2RX1) antagonist, were analyzed in a mouse two-hit model of TRALI. Mice were primed with lipopolysaccharide (LPS) and 24 h later challenged by administrating an anti-MHC I antibody. The selective P2RX1 antagonist NF449 was administrated before the administration of LPS and/or the anti-MHC I antibody. When given before antibody administration, NF449 improved survival while maximal protection was achieved when NF449 was also administrated before the sensitization step. Under this later condition, protein contents in bronchoalveolar lavages were dramatically reduced. Cell depletion experiments indicated that monocytes/macrophages, but not neutrophils, contribute to this effect. In addition, the reduced lung periarteriolar interstitial edemas in NF449-treated mice suggested that P2RX1 from arteriolar smooth muscle cells could represent a target of NF449. Accordingly, inhibition of TRPC6, another cation channel expressed by smooth muscle cells, also reduced TRALI-associated pulmonary interstitial and alveolar edemas. These data strongly suggest that cation channels like P2RX1 or TRPC6 participate to TRALI pathological responses.

## Introduction

Transfusion-related acute lung injury (TRALI) is defined as a non-cardiogenic pulmonary edema occurring during or within 6 hours of blood transfusion^1,2^. TRALI is the most prevalent remaining cause of transfusion-associated morbidity and mortality^3^ and there is no satisfactory therapeutic option^4^. Retrospective studies have shown that anti-HLA I, anti-HLA II or anti-HNA allogeneic antibodies present in the transfused products can trigger TRALI; the involvement of metabolic triggers released during the storage of platelets and/or erythrocytes is debated^5^. An early model of the pathology proposed that two circumstances concur to provoke this syndrome^6^: an inflammatory state of the receiver (first hit) and the transfusion of a blood product containing allogeneic antibodies from the donor and/or storage-derived metabolites (second hit). A one-hit model has also been proposed, postulating that the presence of relatively high amounts of pathogenic triggers could induce TRALI in the absence of adverse clinical conditions. Nevertheless, in practice, transfusions are performed to compensate a pathological state and epidemiologic analyses indicate that the severity of TRALI is correlated with the seriousness of the pre-transfusion disease, supporting the two-hit model^5^.

Experimentally, TRALI can be provoked within minutes in mice of the H-2^d^ MHC haplotype by injecting the anti-MHC I monoclonal antibody (mAb) 34-1-2S. Hematopoietic cells are major effectors of TRALI responses provoked by anti-MHC I antibodies, but the diversity of the experimental conditions (one hit versus two hit model, amount of injected antibodies, genotype of the animals) have resulted in various conclusions about the contributions of the different blood populations. Cell depletion and/or transfer experiments have indicated that neutrophils are either essential^7–9^ or dispensable^10^ for lung edema formation. Other cells participate to TRALI responses like monocytes and/or macrophages^10,11^, while they control neutrophil recruitment in the lungs through MIP2 secretion^11^. TRALI develops similarly in wild type and rag2-deficient mice, indicating that lymphocytes do not have a major impact^9,10^. In contrast, in another mouse model, suppressor T cells or Tregs have been reported to inhibit TRALI through IL-10-dependent pathway(s)^7,12^. In addition, platelets play a critical role^9^ or are dispensable^13^ for the early TRALI-associated responses that lead to lung edema formation.

During acute lung injury, a plethora of stimuli can induce the release of the damage associated molecular pattern adenosine 5′-triphosphate (ATP) from various cell types such as endothelial and immune cells, platelets and/or stressed erythrocytes. Two classes of membrane receptors mediate the effects of ATP, the ligand-gated P2X cation channels and the G protein-coupled P2Y receptors^14,15^. They are expressed in various combinations depending on the cell type, notably by cells participating in vascular homeostasis^15–18^. ATP receptors positively regulate the recruitment and activation of inflammatory cells^19^ or control vascular parameters such as endothelial barrier integrity and hemodynamics^17^. Among these receptors, the P2X1 receptor (P2RX_1_) is expressed on numerous cell types involved in vascular homeostasis and/or immunity, namely arterial smooth muscle cells (SMCs), neutrophils, macrophages and platelets and therefore might control inflammatory processes involved in the pathogenesis of TRALI. We investigated whether this receptor influences the pathogenesis of TRALI and if so, which P2RX_1_^+^ cells could be involved and could potentially represent a biological target relevant to TRALI-associated pathologic responses.

## Materials and Methods

### Reagents

LPS *(Escherichia coli*, serotype 055: B5) was from Sigma-Aldrich. NF449 (4,4′,4″,4″′-(carbonylbis(imino-5,1,3-benzenetriylbis(carbonylimino))) tetrakis-benzene-1,3-disulphonic acid, octasodium salt) and SAR7334 were provided by Tocris Bioscience. Xylazine (Rompun) and ketamine (Imalgene 1,000) were from Bayer and Merial, respectively. Clodronate liposomes (clodronate 5 mg/mL) were purchased from LIPOSOMA. The allogeneic anti-mouse MHC class I (H-2K^d^/H-2D^d^) mAb (clone 34-1-2S, IgG2a) was from e-Biosciences or Bio X Cell and FITC-labeled anti-Ly6C (clone AL-21) from BD Biosciences. Rat anti-mouse Ly6G (clone 1A8), A700-labeled anti-CD45 (clone 30-F11) and A647-labeled anti-CD11b (clone M1/70) were from Biolegend. Anti-CCR2 (clone MC-21) was kindly provided by Dr. Matthias Mack (University of Regensburg, Germany)^20^.

### Mice

Experiments were performed using 8 to 10 week-old male BALB/cByJ mice (JAX collection) purchased from Charles River Laboratories and used within one week after arrival. The mice were housed in individual ventilated cages of the animal facilities of the EFS-Grand Est (agreement No. E67-482-10). Ethical approval for the animal experiments was obtained from the French Ministry of Research, in accordance with the guidelines of the Regional Committee for Ethics in Animal Experimentation of Strasbourg (CREMEAS, CEEA-35).

### Two-event TRALI model

A previously described two-hit model was used^9^ with a few modifications. Male BALB/cByJ mice (8 to 10 weeks old) were primed with an intraperitoneal injection of LPS (0.1 mg/kg) 24 h before being anesthetized (xylazine (20 mg/kg body weight) and ketamine (100 mg/kg body weight)) and challenged with an anti-H-2K^d^ mAb (clone 34-1-2S, 0.5 mg/kg), its isotype control or saline, injected retro-orbitally. The mice were maintained at 37 °C and sacrificed 10 min or 2 h after injection of the anti-MHC I mAb. All experiments were performed in a blinded manner.

### Drug administration and specific cell depletion

NF449 (10 mg/kg in saline) was delivered i.v. 5 min prior to LPS sensitization and again 5 min before anti-MHC I mAb injection. In some experiments, the mice received NF449 either before LPS or before the anti-MHC I mAb. SAR7334 (10 mg/kg in saline) was injected retro-orbitally into isoflurane (Vetflurane) anesthetized animals, 30 min before TRALI experiments. Anti-Ly6G antibody (clone 1A8, 5 mg/kg in saline) was delivered i.v. 24 h prior to LPS. Anti-CCR2 antibody (clone MC-21, 0.4 mg/kg in saline) was injected i.p. and clodronate liposomes (2 mL/kg) were injected i.v., 18 h after LPS administration, i.e., 6 h prior to injection of the anti-MHC I mAb.

Leukocyte counts were determined in whole blood collected into EDTA (6 mmol/L) by severing the tail of anesthetized mice, using a Scil Vet ABC automatic cell counter (Scil Animal Care Company) set to murine parameters. The percentages of peripheral neutrophils and inflammatory monocytes were determined as described in Flow cytometry paragraph, the absolute counts of the cells were deduced.

### Partial oxygen pressure (pO_2_)

The partial oxygen pressure (pO_2_) was measured in blood drawn into a syringe containing heparin anticoagulant (50 U/mL) from the abdominal aortas of anesthetized mice, 10 min after anti-MHC I injection, using a Critical Care Analyser (OMNIS S; Roche Diagnostics).

### Protein content of bronchoalveolar lavages (BALs)

At 10 min or 2 h after induction of TRALI, a ligation was performed to isolate the left lung (used later for immunohistochemistry), the tracheas of sacrificed mice were exposed and cannulated and 1 mL of sterile PBS solution was injected into the right lung. The bronchoalveolar lavages (BALs) were centrifuged at 13,000 × g for 5 min at 4 °C and the supernatants were stored at −80 °C. The protein content of BALs was quantified using a bicinchoninic acid assay (Thermo Fisher Scientific) according to the manufacturer’s instructions with bovine serum albumin (BSA) as the standard.

### Immunohistochemistry

At the end of the experiments, the left lungs of the mice were removed and fixed in 4% paraformaldehyde overnight. The fixed samples were washed in PBS and maintained in 10% and then 20% sucrose solutions, each time for 24 h, before being snap-frozen in optimal cutting temperature (OCT) embedding medium. Sections (8 μm) were stained with hematoxylin and eosin before histological examination. The areas occupied by edema and by the arterioles were quantified using Image J software on images acquired with a Leica DMLB microscope and the ratio was calculated. The investigator was blinded to the various experimental groups.

### Flow cytometry

Briefly, after TRALI experiments, lungs were perfused with PBS via the right ventricle and an incisure was made in the vena cava as an outlet. Lungs were removed *en bloc* and minced with a scalpel. Minced lungs were placed in PBS with 500 U/mL type IV collagenase and 0.02 mg/mL DNase I and were incubated at 37 °C for 30 min. Digested lungs were passed through a 40 μm filter to obtain single cell suspension. After incubation, cells were counted (ADAM Automated Cell Counter, Digital Bio), resuspended in PBS-1% BSA and Fcγ receptors were blocked with FcR blocking reagent (Miltenyi Biotec). Cells were then stained with directly conjugated anti-CD45, -CD11b, -CD11c, -CD24, -Gr1, -CD170, -F4/80 and -MHC class II mAbs to determine the percentages of neutrophils and inflammatory monocytes, respectively. Cell depletions were controlled by flow cytometric analysis of blood cells stained with directly conjugated anti-CD45, -CD115 and -CD11b mAbs, or with anti-CD45 and anti-Ly6C mAbs. Flow cytometric data were acquired on a Fortessa-X20 flow cytometer (BD Biosciences) and the cell populations were analyzed using BD FACSDiva software. All subsets were gated using fluorescence minus one (FMO) analysis.

### Statistical analyses

**Figure 1:**
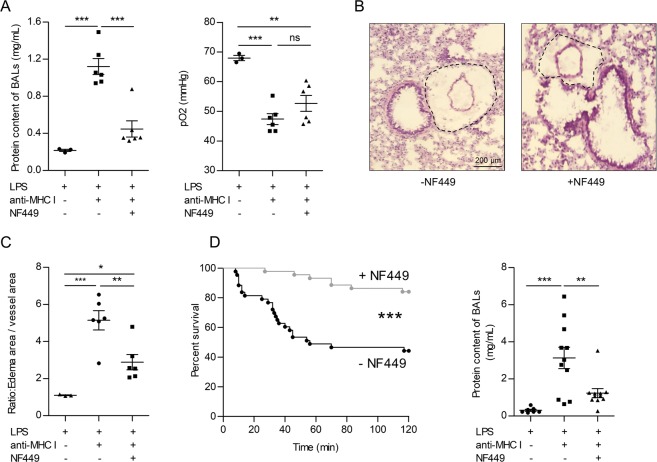
NF449 protects mice against experimental TRALI at early and late stages. NF449 (10 mg/kg, i.v.) was administered prior to LPS injection (0.1 mg/kg, i.p.) and prior to anti-MHC I antibody challenge (0.5 mg/kg, i.v., +) or negative control (−). The pathogenesis of TRALI was analyzed 10 min after anti-MHC I mAb injection **(A,B,C)** or 2 h later **(D)**. **(A)** Left panel: analysis of protein concentrations in BALs using a BCA assay, mean ± SEM (***p < 0.001). Right panel: pO_2_ in aortic blood, mean ± SEM (ns p > 0.05, **p < 0.01, ***p < 0.001). **(B)** Representative lung sections stained with eosin and hematoxylin showing periarteriolar edema. **(C)** Ratio of the area of vessels and periarteriolar edema to that of vessels, mean ± SEM (*p < 0.05, **p < 0.01, ***p < 0.001). **(D)** Left panel: Kaplan-Meier survival plots for mice treated with NF449 or vehicle (saline solution) (n = 20, ***p < 0.0001, log-rank test). Right panel: analysis of protein concentrations in BALs using a BCA assay, mean ± SEM (**p < 0.01, ***p < 0.001).

**Figure 2:**
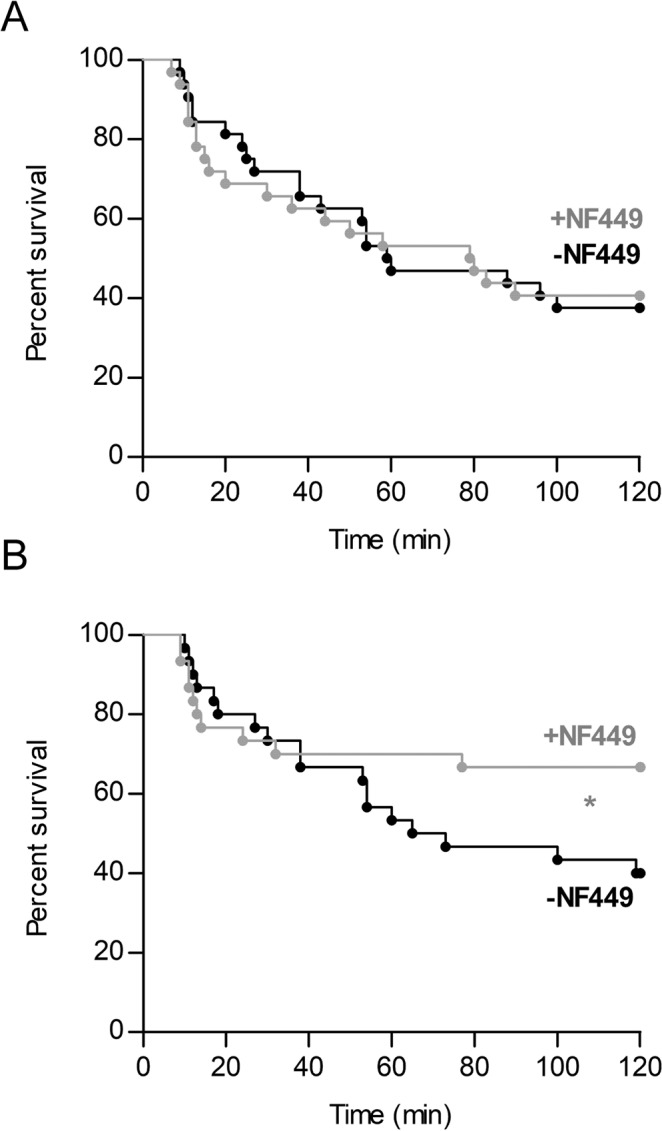
NF449 needs to be administered before each step of experimental TRALI to provide maximal protection. (**A**) Effect of the administration of NF449 (10 mg/kg, i.v.) before LPS injection only (0.1 mg/kg, i.p.). Kaplan-Meier survival plots for mice treated with NF449 or vehicle (saline solution) (n = 30, ns p > 0.05, log-rank test). (**B**) Effect of the administration of NF449 (10 mg/kg, i.v.) before anti-MHC I antibody challenge only (0.5 mg/kg, i.v.). Kaplan-Meier survival plots for mice treated with NF449 or vehicle (saline solution) (n = 32, ns p > 0.05, log-rank test). Statistical analysis of survival at the 2-hour endpoint indicated a significant protective effect of NF449 (n = 32, *p = 0.0384, χ2 test).

**Figure 3:**
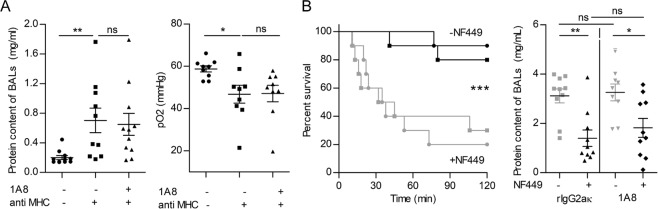
Neutrophil depletion does not prevent the development of TRALI, whereas treatment with NF449 does. **(A)** The mAb 1A8 (5 mg/kg, i.v.), or rIgG2ak, was administered 24 h before LPS (0.1 mg/kg, i.p.) and the pathogenesis of TRALI was analyzed 10 min after anti-MHC I mAb challenge (0.5 mg/kg, i.v., +) or negative control antibody (−). Left panel: protein concentrations in BALs, mean ± SEM (**p < 0.01, ns p > 0.05). Right panel: pO_2_ in aortic blood, mean ± SEM (*p < 0.05, ns p > 0.05). **(B)** The mAb 1A8, or rIgG2ak, was injected 24 h before LPS, NF449 (10 mg/kg, i.v.) was delivered prior to LPS injection and prior to anti-MHC I mAb challenge (0.5 mg/kg, i.v.) and the pathogenesis of TRALI was analyzed 2 h later. Left panel: Kaplan-Meier survival plots for mice treated or not with 1A8 and/or NF449 (***p < 0.001, log-rank test, −NF449 vs +NF449). Right panel: protein concentrations in BALs, mean ± SEM (ns p > 0.05, *p < 0.05, **p < 0.01).

Statistical analyses were performed with GraphPad software (Prism 5.02). Survival studies were analyzed using the log-rank test (Figs 1D, 2A,B and 3B) or the χ2 test (Fig. 2B). Other data are reported as the mean ± SEM and were analyzed using 1- or 2-way ANOVA followed by a Dunn’s test or a Bonferroni post hoc analysis as appropriate. A P-value of <0.05 was considered to be statistically significant.

## Results

### The P2RX_1_ receptor antagonist NF449 decreases the severity of experimental TRALI

To explore the role of P2RX_1_, we used an experimental TRALI model consisting of administration of the anti-MHC I mAb 34-1-2S (anti-H-2K^d^, -H-2D^d^, 0.5 mg/kg) in LPS-sensitized H-2^d^ BALB/c mice, taking advantage of the availability of the selective P2RX_1_ antagonist NF449^21,22^. NF449 displays good selectivity for the P2RX_1_ with respect to other P2 receptor subtypes and potently antagonizes (IC_50_ = 5.8 ± 2.2 µM) the P2RX_1_-mediated calcium influx induced by α,β-methyleneadenosine 5′-triphosphate (α,βMeATP) in murine platelets^22–24^.

BALB/c mice were treated with NF449 (10 mg/kg, i.v.) or vehicle (saline) prior to LPS injection (0.1 mg/kg, i.p.) and anti-MHC I antibody challenge (0.5 mg/kg, i.v.). Ten minutes later, the features of experimental TRALI, such as lung edema and the drop in blood oxygenation, can be measured whereas survival is evaluated within a 2 h period. Lung edema is evaluated by quantifying the proteins in bronchoalveolar lavages and respiratory distress by the measure of the partial oxygen pressure (pO_2_) in aortic blood as previously described^13^. After 10 min, the protein content of BALs was significantly lower in NF449-treated than in saline-treated animals (Fig. 1A, left panel). Accordingly, histological analyses of lung tissues revealed larger areas of interstitial periarteriolar edema in saline-treated mice as compared to those receiving NF449 (Fig. 1B,C), while no edema was observed in the proximity of the post-alveolar microcirculation (Supplementary Fig. 1). Nevertheless, pO_2_ dropped similarly in both groups (Fig. 1A, right panel).

Within the two hours following antibody injection, 56% of the control mice (treated with saline) died, while this percentage fell to 16% in mice receiving NF449 (Fig. 1D, left panel). The protein content of BALs was significantly increased in saline-treated mice challenged with the anti-MHC I antibody as compared to non-challenged mice. On the other hand, treatment with NF449 reduced the protein concentration in BALs by more than two-fold (Fig. 1D, right panel). Altogether, these results indicated that in mice, NF449 reduces the disruption of the endothelial barrier provoked by antibody-mediated TRALI, which allows better survival.

### NF449 needs to be administered before each step of experimental TRALI to provide maximal protection

We then investigated whether NF449 needed to be administered at each step of the two-hit experimental TRALI model, i.e., before LPS sensitization and/or before anti-MHC I mAb challenge, to reduce its pathology. The survival of the animals that received saline or NF449 prior to LPS sensitization only was similar (Fig. 2A). Therefore, administration of NF449 exclusively before LPS sensitization did not improve survival.

In contrast, when NF449 was delivered only before the anti-MHC I antibody challenge, 33% of the mice died as compared to 60% of saline-receiving animals (Fig. 2B). Interestingly, whereas the whole survival curves of saline- and NF449-treated mice revealed no significant differences, survival at the 120 min endpoint was significantly improved in those receiving NF449 (n = 32, *p = 0.0384, χ2 test). Thus, treatment with NF449, before antibody administration only, improved survival, while maximal reduction of the severity of experimental TRALI required administration of NF449 also before the sensitization step.

### The protective effect of NF449 does not rely on neutrophils and inflammatory monocytes

Subsequently, we evaluated the contribution of P2RX_1_^+^ immunological cells to TRALI, namely neutrophils, monocytes and macrophages. Neutrophils can be selectively depleted for at least three days by treatment with the anti-Ly6G mAb 1A8^25^. This antibody was administered 24 h before LPS sensitization and flow cytometric analyses performed 48 h later, before anti-MHC I injection, confirmed their depletion (Supplementary Fig. 2A). The pathology of TRALI at the 10 min time point was similar in 1A8-treated and untreated mice, as assessed by the measure of the protein concentration in BALs and the pO_2_ (Fig. 3A). Immunohistology confirmed that neutrophils could not be detected in the lungs of 1A8-treated mice after TRALI, indicating that even residual circulating neutrophils had not been recruited (Supplementary Fig. 2B).

We next tested whether NF449 still has a protective effect on survival in neutropenic animals over a 2 h time course. In the absence of NF449 treatment, neutrophil depletion did not modify the severity of antibody-mediated TRALI in terms of survival at the 2 h endpoint (Fig. 3B, left panel). In contrast, 80% of mice treated with NF449 survived regardless of the neutrophil count (Fig. 3B, left panel). Accordingly, the protein content of BALs was similarly elevated after TRALI in neutropenic and non-neutropenic mice that did not receive NF449, but significantly decreased in NF449-treated animals (Fig. 3B, right panel). These results indicated that neutrophils do not play a major role in experimental TRALI and hence that other cell types must be responsible for the contribution of P2RX_1_ to TRALI.

**Figure 4:**
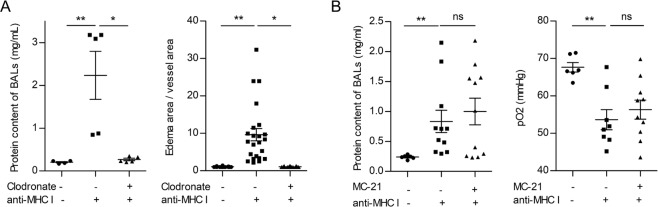
Monocyte/macrophage depletion, but not inflammatory monocyte depletion, prevents the development of TRALI. **(A)** Clodronate liposomes (2 mL/kg, i.v.), or saline, were injected 6 h before the anti-MHC I mAb (0.5 mg/kg, i.v.). Left panel: protein concentrations in BALs, mean ± SEM (**p < 0.01, *p < 0.05). Right panel: ratio of the area of interstitial edema to the vessel area, mean ± SEM (**p < 0.01, *p < 0.05). **(B)** MC-21 (0.4 mg/kg, i.p.), or rIgG2bκ, was injected 6 h before anti-MHC I mAb challenge (0.5 mg/kg, i.v.). Left panel: protein concentrations in BALs and right panel, pO_2_ in aortic blood, mean ± SEM (**p < 0.01, ns p > 0.05).

We then used clodronate liposomes, which eliminate all types of monocytes and also macrophages. To allow inflammation to proceed, the liposomes were delivered 18 h after LPS, i.e., 6 h before anti-MHC I mAb injection. Flow cytometric analyses confirmed that this procedure depleted tissue macrophages and peripheral monocytes (Supplementary Fig. 3A,B). Under these conditions, antibody-mediated TRALI was totally abolished. Indeed, 10 min after anti-MHC I administration, the protein content of BALs was dramatically reduced in clodronate-treated mice as compared to control animals (Fig. 4A, left panel) and no interstitial edema was observed on histological preparations (Fig. 4A, right panel).

We then attempted to evaluate the specific contribution of monocytes. Monocytes can be divided into two main subtypes, the patrolling and the inflammatory monocytes. The former weakly express Ly6C and are CCR2^−^, whereas the latter are Ly6C^+^ and CCR2^+^ and represent the major subclass of blood monocytes (80% of monocytes in BALB/c mice). This pattern allows the depletion of inflammatory monocytes, whereas there is no available treatment to specifically deplete patrolling monocytes in BALB/c mice. Administration of the anti-CCR2 mAb MC-21, 18 h after LPS treatment, i.e., after the maximum concentration of inflammatory cytokines in the blood had been reached^26^, resulted in the depletion of inflammatory monocytes (Supplementary Fig. 2C), in accordance with previous studies^20^. This cell specific depletion did not modify the development of TRALI triggered 6 h later, as assessed by measurement of the protein content of BALs (Fig. 4B, left panel) and the aortic pO_2_ (Fig. 4B, right panel). We could therefore conclude that inflammatory monocytes are dispensable for the initiation of experimental antibody-mediated TRALI.

Altogether, these findings strongly indicate that activation of patrolling monocytes and/or macrophages triggers the pathology of TRALI and that their P2RX_1_ could contribute to TRALI.

### The protective effect of NF449 is consistent with a contribution from P2RX_1_ expressed on lung periarteriolar smooth muscle cells (SMCs)

The development of pulmonary periarteriolar edemas during TRALI early biological responses might be driven in part by the pulmonary arterial pressure. The blood pressure in the lung pre-capillary arterioles is controlled by SMCs. Contraction of these cells is positively regulated by P2RX_1_ present on their surface^27^ and their deficiency prevents the development of pulmonary arterial hypertension^27^. Thus, the reduced edema areas in NF449-treated mice could be due to the inhibition of periarteriolar SMC contraction. Interestingly, inhibition of another cation channel, the TRPC6 calcium channel, which is expressed by pulmonary SMCs, also inhibits pulmonary arterial hypertension and the associated edema during acute lung injury^28^.

**Figure 5:**
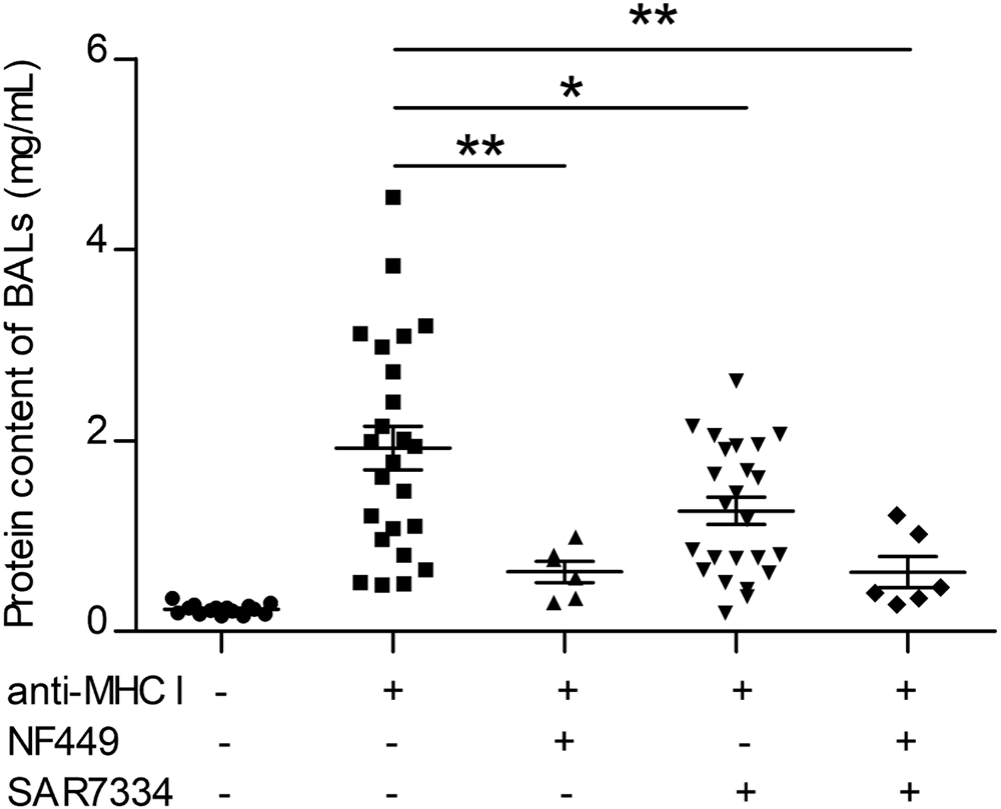
The TRPC6 inhibitor SAR7334 decreases the extent of lung edema. NF449 (10 mg/kg, i.v.) was administered 5 min prior to LPS injection (0.1 mg/kg, i.p.) and 5 min prior to anti-MHC I antibody challenge (0.5 mg/kg, i.v., +) or negative control (−). SAR7334 (10 mg/kg, i.v.) was administered 30 min prior to anti-MHC I antibody challenge. Protein concentrations in BALs, mean ± SEM (*p < 0.05, **p < 0.01).

We therefore tested the effects on TRALI of SAR7334, a specific inhibitor of TRPC6. SAR7334 was administered i.v. 30 min before the anti-MHC I mAb and TRALI responses were analyzed 10 min later. Remarkably, the quantity of proteins in BALs was reduced in animals receiving the inhibitor (Fig. 5). Histological analyses of the lungs revealed that the extent of periarteriolar edema was diminished after SAR7334 treatment (Supplementary Fig. 4). Thus, inhibition of the TRPC6 calcium channel expressed by SMCs reduced the formation of TRALI-associated lung edema. Combined administration of NF449 and SAR7334 did not however further inhibit TRALI-associated lung edema (Fig. 5). These findings strongly suggest that the P2RX_1_ present on lung periarteriolar SMCs could also contribute to the protective effect of NF449.

## Discussion

Our present study shows that NF449 reduces the severity of TRALI in mice, indicating a major contribution of the P2X_1_ receptor. Maximal reduction of the experimental TRALI features required treatment with NF449 before the sensitization step with LPS and before administration of the anti-MHC I mAb. Of note, NF449 treatment before injection of the anti-MHC I mAb only, also resulted in a significant improvement in the survival of the animals.

P2RX_1_ appeared to control different mechanisms at each step of the two-hit model. LPS responsive cells, namely monocytes or macrophages, which express P2RX_1_^29^, were most likely the primary targets of NF449 in the first hit, although the role of P2RX_1_ on these cells during LPS sensitization remains to be clarified. In the second step, the protective effect of NF449 could be related to all cell types expressing the P2RX_1_ and participating in vascular homeostasis. Endothelial cells (ECs) and platelets were not considered, since ECs do not express P2RX_1_^30^, while platelets are dispensable for the initiation of TRALI^13^. We first explored the role of neutrophils since P2RX_1_ has been shown to control neutrophil responses by amplifying chemotactic signals^31,32^. Here, their immunodepletion did not modify the features of experimental TRALI at the 10 min or 2 h time points.

This absence of a major role of neutrophils may appear to be in conflict with the accepted view of their importance in TRALI. However, in the literature, part of the evidence supporting the contribution of neutrophils to TRALI responses is indirect. Firstly, in man, post mortem histological analyses have shown that TRALI is accompanied by the recruitment of neutrophils to the lungs, but these observations cannot indicate whether this neutrophil recruitment is the result or the cause of TRALI initiation^33^. Anti-HLA class I antibodies could nevertheless induce TRALI in a neutropenic receiver (less than 0.1 10^9^ cells/L)^34^. Secondly, in animal models, neutrophils are ideal candidate cells based on their recruitment to the lungs and their ability to produce damaging reactive oxygen species *in vitro*, while conditions inhibiting this production protect the animals from developing TRALI^11,35–39^.

Direct evidence for the requirement for neutrophils in antibody-mediated TRALI exists, but can be debated. It is based on *in vivo* studies using transfer of isolated bone marrow neutrophils^8^, which may not be representative of circulating neutrophils since it is technically difficult to isolate these cells in a resting state, or on cell depletion in animals followed by intratracheal instead of intraperitoneal administration of LPS^9^, which produces fundamentally different inflammatory conditions. Indeed, intratracheal administration of LPS leads to neutrophil recruitment to the alveoli, whereas intraperitoneal injection of low doses of LPS, as in our work, does not. A second team reported that neutrophil depletion blocked lung edema formation^7^, while a third failed to observe any protective effect^10^. It is difficult to compare our observations with those of these two groups, since the experimental conditions were very different: (i) LPS sensitization in our study *versus* no sensitization in the two others (two-hit *versus* one-hit model), (ii) different doses of anti-MHC I antibody (0.6 mg/kg in our study *versus* 5 to 50 mg/kg), (iii) variable breeding conditions (breeder or animal facility environment) and (iv) different genetic backgrounds (BALB/cByJ, BALB/cAnNCrl, BALB/cAnNTac). Notably, with respect to the last two points, BALB/cByJ and BALB/cAnNCrl mice have been reported to display a different susceptibility to Theiler’s murine encephalomyelitis virus-induced demyelinating disease^40^, and the commensal microbiota may affect TRALI responses^41^. Nonetheless, we do not exclude that neutrophils could participate in later TRALI responses such as extravasation, which requires P2RX_1_ activity^32^.

We showed that clodronate liposomes, which eliminate monocytes, macrophages and dendritic cells (DCs), protected mice from TRALI. On the other hand, immunodepletion of CCR2^+^ inflammatory monocytes did not influence the development of TRALI, indicating that inflammatory monocytes are not key players in this syndrome. How and which of these cells contribute positively to TRALI remains to be elucidated. Distal Kupffer cells or splenic macrophages could participate in TRALI, as observed in other ALI models^42^, or more likely arteriolar macrophages^43^ or dendritic cells^44^, given the localization of the interstitial lung edema. Administration of diphtheria toxin to transgenic animals expressing its receptor on CD11c^+^ cells, which include DCs, exacerbates TRALI-associated lung damage^7^. However, this experimental set-up can lead to neutrophilia^45^, which could represent a confounding effect in this neutrophil-dependent TRALI model. Thus, complementary experiments will be required to clarify the role of DCs, at least in our experimental model.

Remarkably, interstitial lung edema was observed in the pre-microcapillary arteriolar spaces but not in the post-alveolar circulation. This localization is consistent with the observation that ALI begins with periarteriolar edema which, after exceeding a threshold, floods into the alveoli^46^. Accordingly, real time analyses of the development of lung edema in pigs^47^ or in man have shown that the extravascular lung water volume increases before a defect in blood oxygenation is ascertained^48^. These facts strongly suggest that the arteriolar tissue is the primary site of the edematous process during anti-MHC I-induced TRALI. The reduced lung periarteriolar interstitial edemas in NF449-treated mice suggested that P2RX_1_^+^ vascular SMCs could participate in the formation of interstitial lung edema by promoting arteriolar vasoconstriction and thereby a transient increase in pulmonary blood pressure, which would favor plasma leakage^46^. Stimulation of these cells induces vasoconstriction in a P2RX_1_-dependent manner, resulting in pulmonary arterial hypertension, which is in part responsible for edema formation^27^. We note that in a rat model of TRALI, transfusion of the plasma from aged erythrocyte concentrates was accompanied by an increase in pulmonary arterial blood pressure^6^. Accordingly, in two cases of human TRALI observed after the transfusion of blood products containing anti-HLA class I antibodies, a rise in pulmonary arterial tension was detected before the onset of hypoxemia and acute respiratory distress^49,50^. TRPC6 is another calcium channel expressed by SMCs, which positively controls vasoconstriction, pulmonary arterial hypertension and edema formation^28,51^. Remarkably, we showed that the TRPC6 inhibitor SAR7334 also reduced the accumulation of proteins in BALs and the extent of the periarteriolar edema provoked by TRALI, which could represent the first example of a protective effect *in vivo* of a specific TRPC6 inhibitor^28^. The expression profiles of TRPC6 in the hematopoietic lineage indicate that murine monocytes and macrophages do not significantly express this gene (heamosphere.org and immgen.org), while TRPC6 is expressed by ECs. Therefore, we cannot formally exclude that inhibition of endothelial TRPC6 also contributes to the protective influence of SAR7334.

Currently, in the case of TRALI, the mainstay of treatment remains general supportive care, including fluid resuscitation, oxygenation or mechanical ventilation^52^. Recent studies have indicated that the administration of IL-10 or CRP inhibitors could represent promising therapeutic or prophylactic strategies^53^. Our findings now suggest that targeting cation channels like P2RX_1_ or TRPC6, by reducing interstitial lung edema, could constitute an additional pharmacological approach for TRALI management, given that intensive medical care lasts several hours.

## Supplementary information

Supplementary figures
