# Supplementary material for: The ATP-gated P2X1 ion channel contributes to the severity of antibody-mediated Transfusion-Related Acute Lung Injury in mice
Source: Sci Rep. 2019 Mar 26;9:5159. doi: 10.1038/s41598-019-41742-9 (PMC6435740; doi:10.1038/s41598-019-41742-9)
Supplement: Supplementary file 1 — Supplementary figures [file 41598_2019_41742_MOESM1_ESM.pdf]

Title:

The ATP-gated $P2X_1$ ion channel contributes to the severity of antibody-mediated Transfusion-Related Acute Lung Injury in mice

Authors: Marie-Belle EL MDAWAR*, Blandine MAÎTRE*, Stéphanie MAGNENAT, Christian GACHET, Béatrice HECHLER[§], Henri DE LA SALLE[§]

- Both authors contributed equally to the work

[§] These authors share senior authorship

Attributions: Université de Strasbourg, INSERM, Etablissement Français du Sang (EFS) Grand Est, BPPS UMR_S 1255, Fédération de Médecine Translationnelle de Strasbourg (FMTS), F-67000

Address for correspondence: UMR_S1255 INSERM, Université de Strasbourg, Etablissement Français du Sang-Grand Est, 10 rue Spielmann, BP 36, F-67065 Strasbourg Cedex, France

email: beatrice.hechler@efs.sante.fr; henri.delasalle@efs.sante.fr

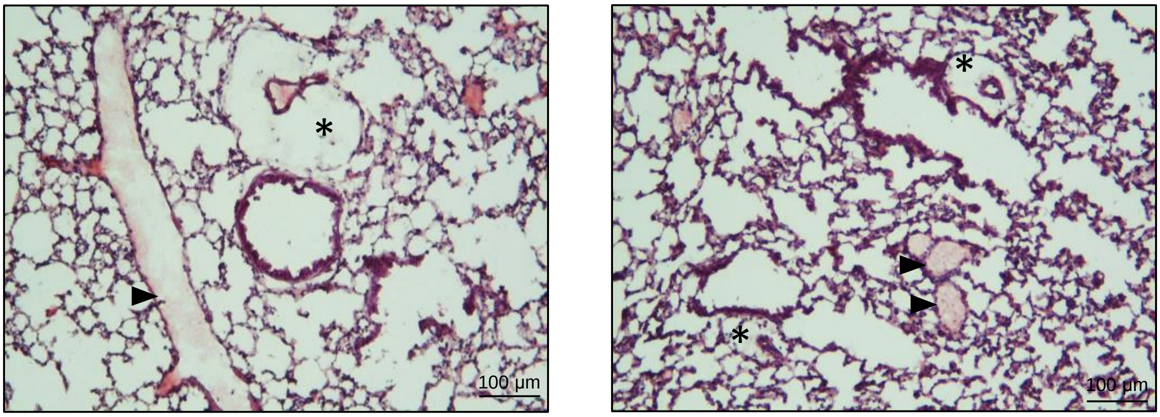

Supplementary Figure 1: Interstitial edema was not observed in the proximity of post-capillary venules after TRALI.

TRALI was induced by injection of 34-1-2S and 10 min later, the lungs were recovered, fixed and processed for histology. Two representative views of sections are shown. Periarteriolar interstitial edema (*) and post-capillary venules/veins (arrow heads).

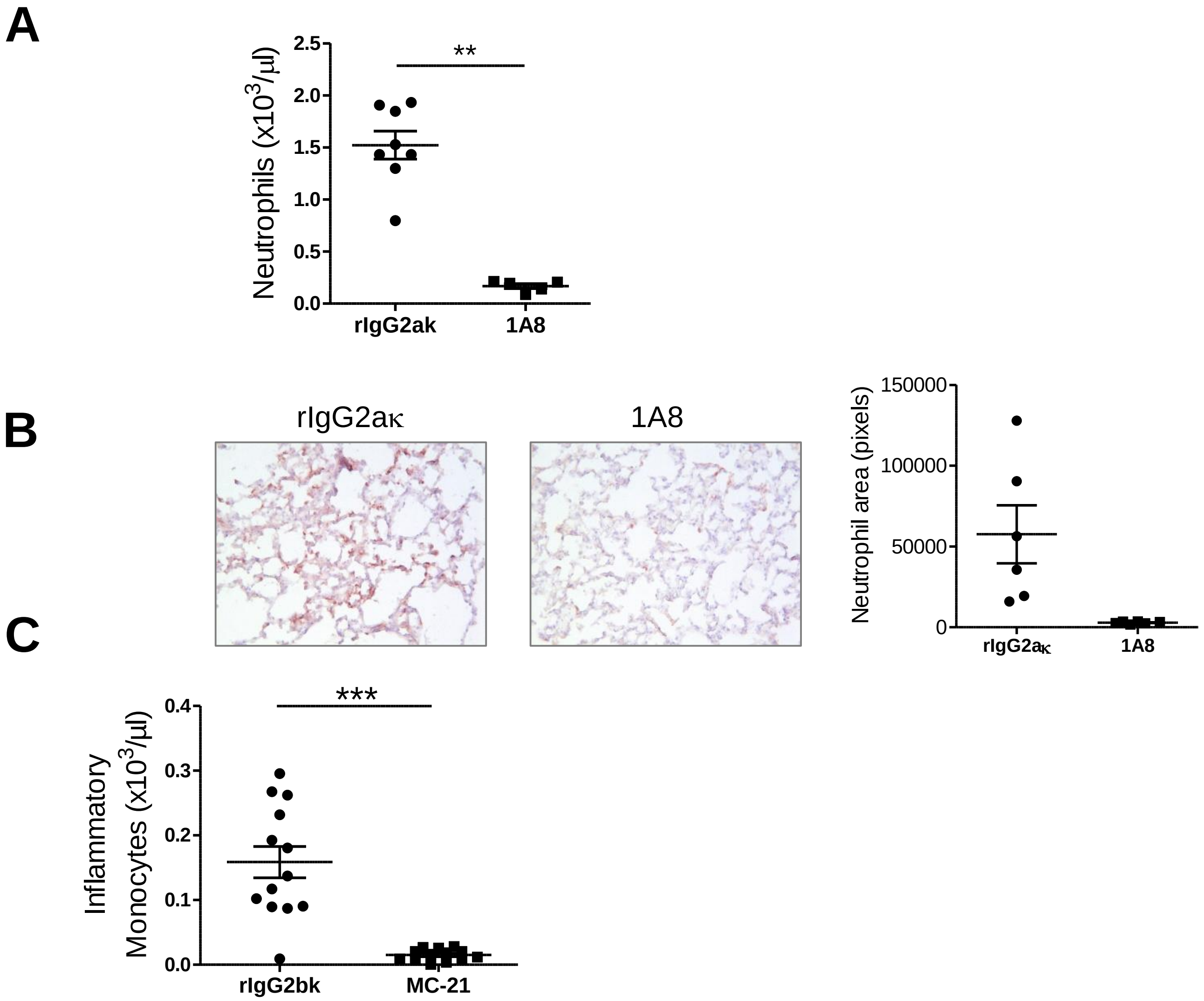

Supplementary Figure 2: Confirmation of specific cell depletions.

Leukocyte counts in peripheral blood and the percentages of $CD45^+$ $CD11b^+$ $CD115^-$ neutrophils and of CD45+ $Ly6C^{bright}$ inflammatory monocytes among $CD45^+$ leukocytes were determined as described in the method section. **(A)** Analysis of neutrophil depletion after 1A8 treatment (5 mg/kg, i.v.) and before anti-MHC I mAb injection. The histogram represents the absolute counts of peripheral neutrophils, mean ± SEM (n=8 for -1A8 and n=5 for +1A8, ** p<0.01). **(B)** Histochemical analysis of lung neutrophils in mice, 10 min after administration of 34-1-2S mAb, in 1A8- or negative control (rIgG2aκ)-treated mice. **(C)** Analysis of inflammatory monocyte depletion after MC-21 treatment (0.4 mg/kg, i.p.) and before anti-MHC I mAb injection. The histogram represents the absolute counts of inflammatory monocytes, mean ± SEM (n=13, *** p<0.001).

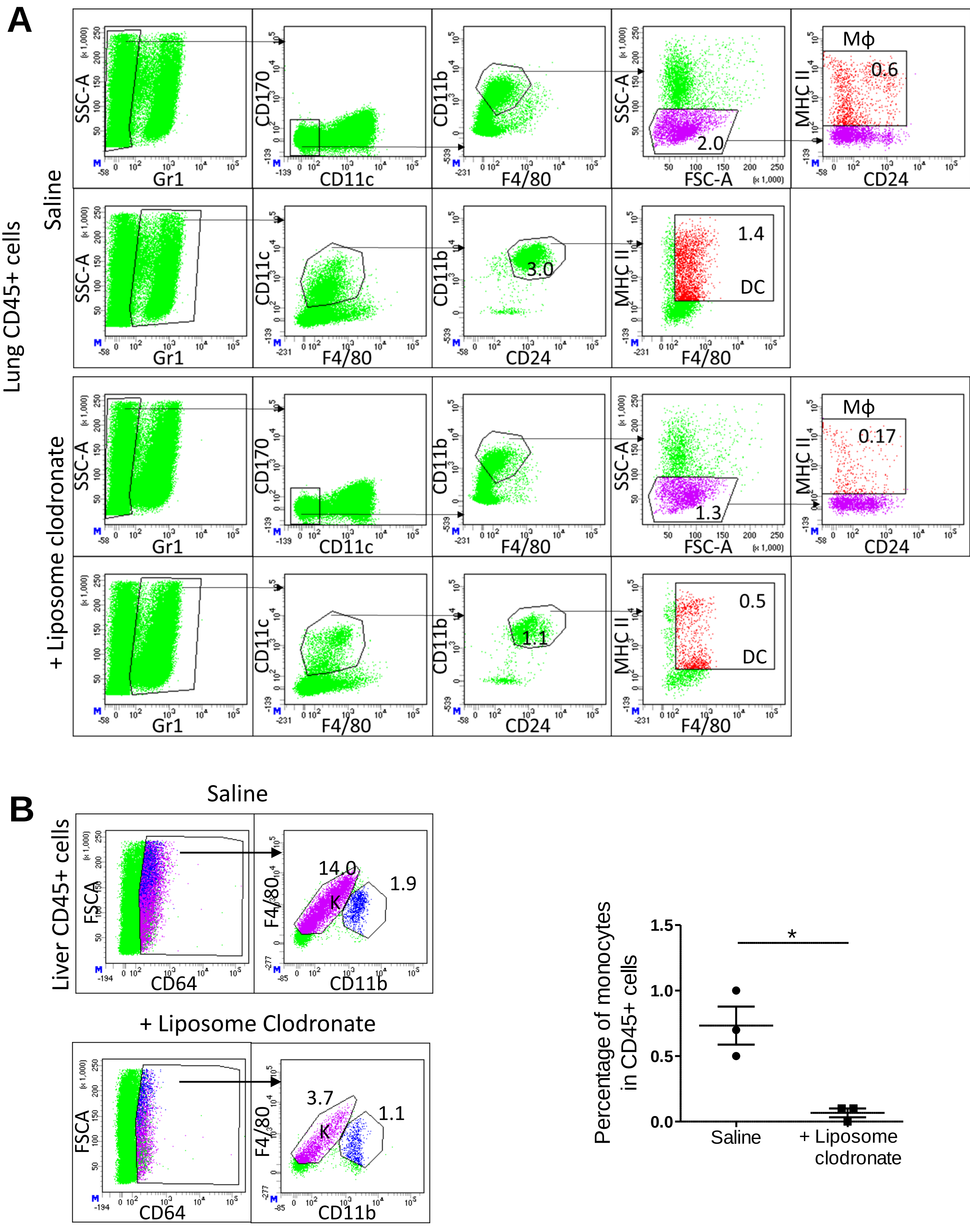

Supplementary Figure 3

Supplementary Figure 3: Effects of *in vivo* clodronate liposome treatment on lung and liver cells.

Representative schemes of flow cytometric analysis of lung and liver macrophages and/or dendritic cells 6 h after the administration of a suspension of clodronate liposomes (2 mL/kg, i.v.) or alternatively, saline. (**A)** Interstitial macrophages (Mϕ) are defined as Gr-1- $CD11b^+$ $CD24^-$ $CD170^-$ $CD11c^-$ MHC II^+ $F4/80^+$ cells and dendritic cells (DC) as Gr-1^+ $CD11b^+$ $CD24^+$ $CD11c^+$ MHC II^+ $F4/80^+$ cells. **(B)** Left panel: $CD64^+$ liver macrophages can be divided into $CD11b^{dim}$ clodronate-sensitive Kupffer cells (K) and clodronate insensitive $CD11b^{bright}$ subpopulations. Numbers refer to the percentages of cells among viable $CD45^+$ cells in each organ, mean ± SEM (n=4). Right panel: Percentage of monocytes ($CD11b^+$ $CD115^+$) among viable $CD45^+$ cells in saline and clodronate liposomes-treated mice, mean ± SEM (n=3, * $p<0.05$).

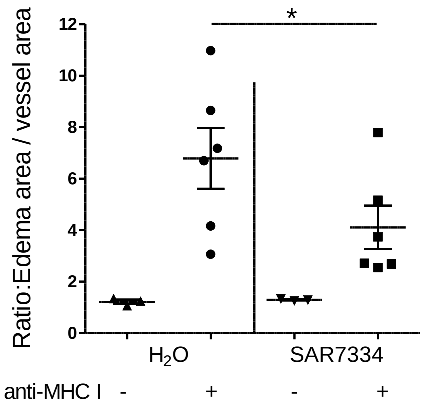

Supplementary Figure 4: The TRPC6 inhibitor SAR7334 decreases the extent of lung edema.

LPS-sensitized mice received SAR7334 (10 mg/kg) and 30 min later, the mAb 34-1-2S (0.5 mg/kg). Animals were sacrificed after 10 min and lung were sampled for further histologic analysis. Ratio of the area of vessels and periarteriolar edema to that of vessels, mean ± SEM (* $p<0.05$).
